# Progressive and instantaneous nature of lithium nucleation discovered by dynamic and operando imaging

**DOI:** 10.1126/sciadv.adg6813

**Published:** 2023-05-24

**Authors:** Guangxia Feng, Yaping Shi, Hao Jia, Samprash Risal, Xu Yang, Paul Ruchhoeft, Wei-Chuan Shih, Zheng Fan, Wu Xu, Xiaonan Shan

**Affiliations:** ^1^ Department of Electrical and Computer Engineering, University of Houston, Houston, TX 77204, USA.; ^2^ Energy and Environment Directorate, Pacific Northwest National Laboratory, Richland, WA 99354, USA.; ^3^ Department of Engineering Technology, University of Houston, Houston, TX 77204, USA.

## Abstract

The understanding of lithium (Li) nucleation and growth is important to design better electrodes for high-performance batteries. However, the study of Li nucleation process is still limited because of the lack of imaging tools that can provide information of the entire dynamic process. We developed and used an operando reflection interference microscope (RIM) that enables real-time imaging and tracking the Li nucleation dynamics at a single nanoparticle level. This dynamic and operando imaging platform provides us with critical capabilities to continuously monitor and study the Li nucleation process. We find that the formation of initial Li nuclei is not at the exact same time point, and Li nucleation process shows the properties of both progressive and instantaneous nucleation. In addition, the RIM allows us to track the individual Li nucleus’s growth and extract spatially resolved overpotential map. The nonuniform overpotential map indicates that the localized electrochemical environments substantially influence the Li nucleation.

## Teaser

A dynamic and operando imaging technique reveals the progressive and instantaneous nature of lithium nucleation.

## INTRODUCTION

Lithium (Li) metal is the ideal anode material due to its ultrahigh theoretical specific capacity and the lowest electrochemical redox potential (*1*–*3*). The industrial application of Li metal batteries (LMBs) has been hindered by the poor cycling life, low Coulombic efficiency, and critical safety concerns (*4*, *5*) due to the instability and inhomogeneity of the Li deposition and the formation of dendrites during the continuous battery operation process (*6*–*8*). The “anode-free” configuration is one of the most promising configurations that could make the battery reach the goal of 500 Wh kg^−1^ possible (*9*, *10*). During the charging process, the potential of the copper (Cu) foil is progressively decreased until it reaches the overpotential of the Li nucleation (*11*–*15*). The Li ions will then diffuse through the solid electrolyte interphase (SEI) and be reduced to the initial Li nuclei. Nonuniform Li growth can cause substantial volumetric expansion of the Li metal anode, which can subsequently break the protective SEI that covers the electrode surface, leading to electrolyte consumption, exacerbating the uneven electric filed distribution and hence aggravating the dendrites formation (*5*, *16*–*18*). Therefore, the capability of studying Li deposition and growth mechanisms is essential to provide a fundamental understanding of the Li deposition process to improve the lifetime and safety of Li metal-based energy storage devices.

Tremendous efforts have been made to develop advanced analytical techniques, including scanning electron microscopy (SEM) (*19*–*23*), transmission electron microscopy (TEM) (*24*–*27*), and atomic force microscopy (AFM) (*28*–*31*), aiming to provide critical information to understand the emergence and evolution of Li nucleation. However, the high-vacuum environment in SEM and TEM makes them difficult to characterize Li nucleation in liquid electrolytes, and the imaging is mostly done after the deposition has already occurred. X-ray–based techniques, including transmission x-ray microscopy (TXM) (*32*–*34*), operando x-ray imaging (*35*) and x-ray computed tomography (*36*–*39*), have been adopted to study the Li plating and stripping processes, which provide detailed insights into the electrode structure and allow us to reconstruct the three-dimensional morphology evolution during Li plating. Nonetheless, the high-energy x-ray beam might interfere and affect the early-stage Li nuclei. Because of the strong transmission, TXM has the unique capability of characterizing commercial battery cells with limited spatial resolution, but the access to a synchrotron imaging beamline further restricts its widespread application in battery studies. AFM is promising for observing the initial stage of Li deposition with high spatial resolution. However, the relatively slow scan rate can limit its temporal resolution, which could lead to the loss of critical information at the very initial nucleation stage. Operando Raman spectroscopy has been widely used to study the Li^+^ intercalation into cathode materials and visualize ion transport during Li deposition (*40*–*42*). However, it is mostly focused on providing reaction’s chemical information rather than Li particles’ growth evolution.

In this work, we used an operando reflection interference microscope (RIM) for real-time imaging of the entire Li nucleation dynamics at single nanoparticle level. The RIM imaging system is built with high sensitivity and high temporal resolution on a Li^0^ half-cell that simulates the “anode-free” LMB. In our previous study, RIM was used to probe the SEI formation dynamics and provide critical insights into the morphological and structural features of SEI (*43*–*45*). The RIM also allows us to image and track the individual Li nuclei’s sizes and locations continuously throughout the deposition and stripping processes and accurately correlate the obtained images to the half-cell’s potentials at any time. Using the RIM, we find that the formation of the initial Li nuclei is not at the exact same time point. This contradicts with the classical theory of instantaneous Li nucleation (*12*, *15*, *46*, *47*), which refers to the formation of nuclei on the electrode surface only at an instance. Our data show that the Li nuclei gradually form on the electrode surface in the early-stage of Li growth process, ~20 s after the electrode reaches its minimum overpotential. The Li nuclei density increases linearly with time in this period, which is the property of progressive nucleation. After this stage, the Li nuclei’s size increases collectively, and the total number of Li nuclei is relatively stable, which is characteristic of the instantaneous nucleation process. These results indicate that the Li nucleation process has both progressive and instantaneous nucleation nature.

By tracking the evolution of individual Li nucleus’ size, we find that both deposition and stripping of Li show two distinct stages. For Li deposition, the first stage is correlated to the Li nuclei formation and growth until they cover almost the entire electrode surface. The second stage is related to continuous Li growth. In the stripping, we also find that the process includes the dissolution of “freshly” deposited Li, which is much faster, and the removal of “old” Li that is close to the electrode surface and mixed with the SEI, which is much harder. Note that the capability of RIM to continuously monitor the Li nucleation and growth process at the same location cannot be easily achieved by other imaging techniques, such as SEM and TEM, which only provide a snapshot of Li nuclei distribution at certain time point.

The formation of the initial Li nuclei is not synchronized. RIM characterization shows that different Li particles start the nucleation at different time points and grow at different speeds, which strongly indicates the inhomogeneity of surface electrochemical environments that considerably affect the Li nucleation and growth. During the charging process, the nonuniform localized electrochemical environments, including the local ion concentration and heterogeneous SEI thickness and quality, dictate the local overpotential and therefore lead to nonuniform Li nucleation. To further illustrate this effect, the localized overpotential map is extracted using the particle size dynamics obtained from the RIM and the Barton’s model (*11*). The distribution of overpotentials at different locations reflects the heterogeneity of localized electrochemical environments. The real-time visualization of Li nucleation dynamics and the localized overpotential map achieved in this work provide important information for the battery interphase design.

## RESULTS

### Using RIM to image Li nucleation

**Fig. 1.**
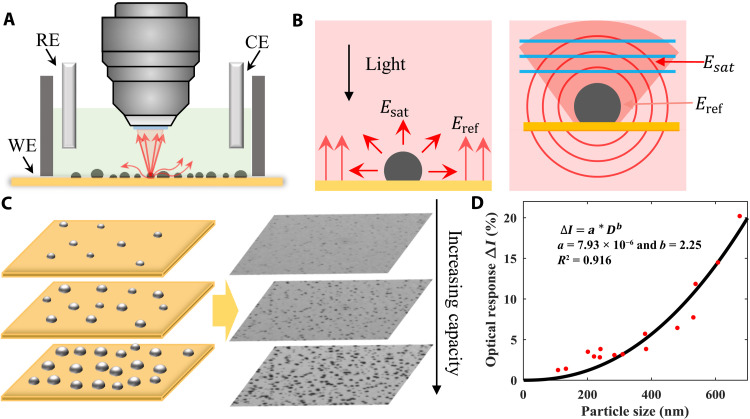
Principle for the RIM imaging system and theory for Li nucleation. (**A**) Schematic for in situ reflection interference microscope (RIM) system. WE, working electrode; CE, counter electrode; RE, reference electrode. (**B**) Interference between the scattering light from particle and the reflection light from substrate. (**C**) Left: Cartoon schematic for lithium (Li) nucleation process. Right: The optical imaging of nucleation process. The formed Li nuclei appear as black dots on the substrate due to the scattering of light. (**D**) Conversion curve that translates the optical signal to particle size. *R*^2^, coefficient of determination.

When the Cu electrode is negatively polarized versus Li/Li^+^, the Li ions diffuse through the electrolyte/SEI layer, the SEI, and the SEI/Cu layer and are reduced on the Cu electrode surface. The reduction of the Li ions leads to nuclei formation (*4*, *48*). To image the evolution of Li nuclei on the electrode surface, a three-electrode system is used (Fig. 1A), where the Cu substrate works as the working electrode (WE), and Li foils are used as the counter and reference electrodes (CE and RE, respectively). During the Li deposition/stripping processes, the individual Li nucleus will scatter the incident light, which will then interfere with the light that is reflected from the Cu electrode surface (Fig. 1B). The interference between the scattered and the reflected light noticeably enhances the contrast and forms the dark spots that are correlated to the Li nuclei (see more details in section S1 and fig. S1) (*49*). When the Li nucleus grows bigger, the scattered light intensity increases, and the corresponding optical spot on the image becomes darker (Fig. 1C, right). In this measurement, the electrolyte of 1 M LiPF_6_/propylene carbonate (PC) was used. In addition, the electrode surface has been carefully cleaned before each experiment (see Materials and Methods for more information).

To establish the correlation between the sizes of the Li nuclei and the dark spots’ intensity, we have imaged the same particles using RIM and SEM (*50*). Note that to minimize the oxidation of Li nuclei, a multifunctional sample box, which will protect the sample with argon (Ar) and transfer the samples from the glove box to the SEM without exposure to any air, has been used to transfer the samples. Figure S2 (A to D) shows the Li nuclei on the RIM image (top) and the corresponding SEM image (bottom). The particle sizes were accurately measured from the SEM images, and the corresponding optical responses were extracted from the RIM images. The correlation between the particle sizes and the RIM responses was extracted using a power law fitting curve in Fig. 1D (see section S2 and fig. S2 for details). In addition, the calibration equation is shown below

$$\text{Δ}I=a\;\cdot D^{b}$$ (1)

where *D* is the diameter of the nanoparticles, and *a* = 7.93 × 10^−6^ and *b* = 2.25 are extracted from Fig. 1D. From this calibration curve, we will be able to extract the Li nucleus size at any time point and location.

**Fig. 2.**
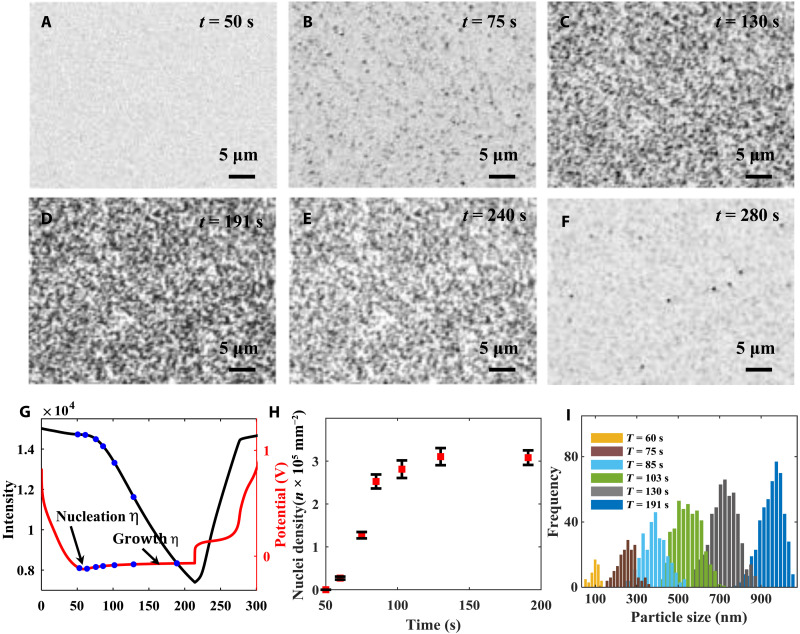
Li nucleation morphology evolution after different amount of deposition capacity. (**A** to **F**) The morphologies of nuclei after (A) 50-, (B) 75-, (C) 130-, and (D) 191-s deposition and (E) 20- and (F) 60-s stripping at a constant current density of 0.1 mA cm^−2^. (**G**) The potential (red curve) and optical signal (black curve) responses during the entire deposition and stripping processes. (**H**) The areal density of nucleated particles at different time points is marked with blue dots in (G). (**I**) Histograms of Li particle sizes after different amount of deposition time.

The localized electrochemical environments will strongly influence the Li nucleation process and lead to a diverse nucleation dynamic, including different nucleus initiation time and growth rate. By using a synchronization device (see section S3 for details), RIM can monitor the entire Li deposition and stripping process, and therefore, we can accurately correlate the images with the corresponding potentials and time stamps (Fig. 2G). Figure 2 (A to F) shows a serial snapshots of Li deposition and stripping video (movie S1) at different time points. Before the potential reaches the minimum (nucleation overpotential, pointed by black arrow in Fig. 2G), there is no Li nuclei formed on the electrode surface (Fig. 2A). When the potential reaches the nucleation overpotential, Li nuclei start to form on the electrode surface (Fig. 2B). We only see a few dim dark spots in the early-stage Li nucleation (Fig. 2B). As the Li deposition continues, more dark spots appear on the electrode surface, and the spots get much darker as Li continuously deposits on the electrode surface. Before the reversal of the deposition current, the density and the intensity of the dark spots both reach their maximum level (Fig. 2D). In the Li stripping process, the Li nuclei start to dissolve, and the intensity of the dark spots decreases (Fig. 2E). This indicates the shrinkage of the Li particles. At the end of the cycle, most of the Li nuclei have been dissolved. However, we can still see some Li particles left on the electrode surface (Fig. 2F), which is partially due to the loss of connection with the electrode.

**Fig. 3.**
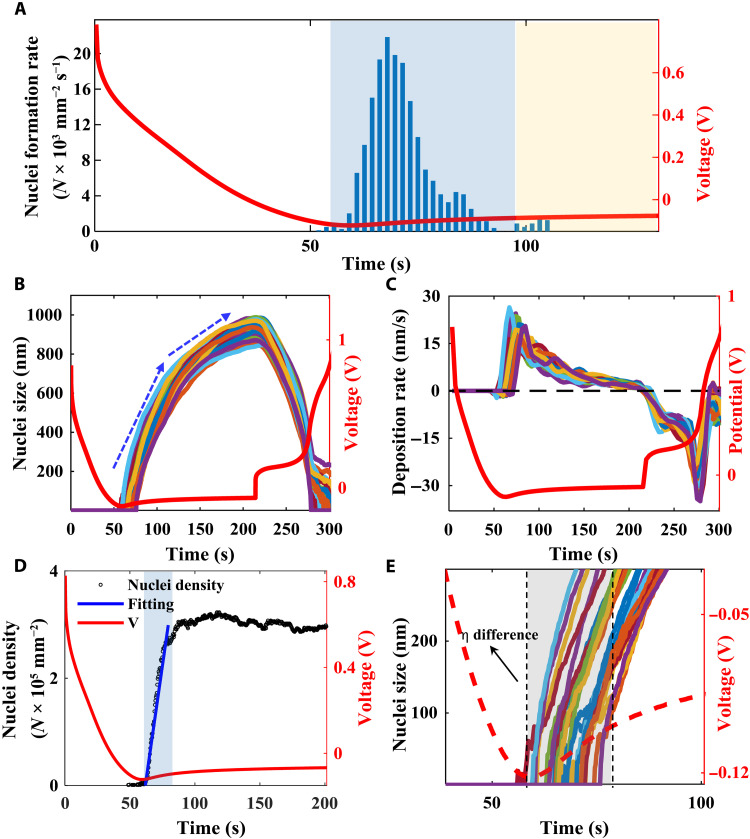
Li nucleation growth dynamics. (**A**) The very initial nuclei formation rate evolution (blue bars) along with voltage (red curve). (**B**) Multiple particles’ deposition and stripping dynamics over time (color curves) and the corresponding voltage responses (red curve). (**C**) Nuclei deposition and stripping rate over time (color curves) and the corresponding voltage response (red curve). (**D**) Li nuclei density evolution (black dots) along with deposition time and potential (red curve). Blue background marks the early-stage progressive nucleation formation, and blue solid line represents the fitting curve of the progressive process with *N*_0_ ≈ 3.14 × 10^5^. (**E**) The nucleation potential variations for different Li nuclei. Colored solid curves indicate the growth of particles along time, red dash curve is the overpotential response, and gray background highlights the overpotential difference for each particle.

RIM has the capability to image the Li nanoparticles that are smaller than 100 nm. Figure S3A shows a snapshot at an early stage of nucleation (at 60 s in Fig. 2H). We can see that individual particle can be clearly separated from the background. The size histogram at this time point is also plotted in fig. S3B, which shows the particles as small as 42 nm. We have also estimated the detection limit of RIM at the current experimental conditions. Figure S1 shows the background noise distribution in RIM setup. A maximum of 0.05% of intensity change (∆*I*) is caused by the random pixel noise. Using the Eq. 1, we estimate that the detection limit of RIM is about 30 nm. In addition, the Fig. 3B also shows that the individual particle’s growth curves and the particle sizes are much smaller than 100 nm at the early stage of the growth. Note that all the curves start from 30 nm, which is the detection limit. In addition, we can further improve the detection limit of our method by increasing the frame rate of the camera. This will allow us average over more images and decrease the pixel noise.

### Imaging Li nucleation formation dynamics

Benefiting from the operando imaging capability and high temporal resolution (up to 1000 frames per second if needed), RIM can image the entire Li nucleation formation and dissolution process at the same location. This capability allows us to track individual Li particles and measure their changes over time and provides us critical information to understand Li nucleation and growth kinetics. On the other hand, the SEM and TEM can only provide a static image at a certain time point, the important dynamic information is missed. Our results show that different Li particles appear at different time points. To further confirm this observation, quantitative analysis in both Li nuclei’s sizes and density is necessary to reveal the relationship between deposition capacity and nucleation dynamics. An algorithm has been developed to identify all the Li nuclei in each frame (see section S5 and fig. S4 for details) and extract the individual particle’s size using the calibration curve (see sections S2 and S6 and fig. S5 for details). Figure 2H shows that the Li nuclei density changes at different deposition capacities and the particle size distribution histogram has also been plotted at different charge capacities. Nuclei density increases initially and stabilizes after it reaches its maximum value. After the maximum nucleation density, the deposition current will generate little new embryos but promote continuous growth of individual nucleus (*12*, *51*). Therefore, we can see that the nuclei density is relatively stable (Fig. 2H), while the particle size increases (Fig. 2I). Furthermore, Li nuclei formed at different current densities were also investigated (see section S7 and fig. S6 for details). The results reveal a positive correlation with the applied current density and the resulted overpotential, which is consistent with the observations by other studies (*12*, *21*, *22*, *30*).

### Li nucleus’ growth and dissolution dynamics at single nanoparticle level

We have characterized each individual Li nucleus’ size and their appearing time. First, we calculate the Li nuclei formation rate at each time point and correlate it with the measured overpotential during the entire deposition process (Fig. 3A). We can see that before the overpotential reaches its minimum value (nucleation overpotential), there is no Li particle formed on the electrode surface. This indicates that the nucleation overpotential (the minimum value of overpotential) has to be reached for Li to overcome the kinetic hindrance of the system. Second, after the nuclear ion overpotential is reached, the nuclei formation rate sharply increases to its maximum value in ~15 s and then quickly drops and lastly stabilizes around zero (Fig. 3A), displaying the overall instantaneous nature of nucleation. This result further verifies our observation in Fig. 2H that, although the nucleation happens in a relatively short time, the nuclei are not formed simultaneously due to the differences in local electrochemical environments.

The derivative of particle size shows the individual Li nucleus’ growth rate in Fig. 3C. For all the Li particles, their growth rates quickly reach the maximum values after the nucleation overpotential, and the growth rates slowly decrease over time (*23*). In the stripping process, the shrinkage rate (negative growth rate) quickly increases to a relatively stable value between 220 and 260 s. Correspondingly, the overpotential turns positive and stays at a relatively stable value as well. In this stage, the freshly deposited Li, which has a large surface area with a poorly developed thin SEI layer, will be dissolved first (*17*). In the second stage, the overpotential increases steeply until it reaches a maximum value. The Li nuclei shrinkage speed further increases and then quickly decreases to near zero. In this stage, the old Li that has a well-developed SEI layer will be dissolved due to the decreased surface area and the relatively thicker SEI layer. The shrinkage rate of these old Li remarkably decreases. The results show that the operando RIM provides a powerful tool to directly observe the reaction dynamics and verify the Li nucleation theory.

Figure 3B shows 25 individual Li nuclei’s size dynamics and the corresponding overpotentials in the Li deposition and stripping processes. We can see that both Li deposition and stripping include two distinct stages. For Li deposition, the first stage (from 50 to 125 s) is correlated to the Li nuclei’s formation and growth until they cover almost the entire electrode surface. The particles’ sizes quickly increase after the overpotential reach its minimum value. The second stage (from 125 to 220 s) is related to the continuation of the Li growth, where Li particles grow in both lateral and vertical directions. The stripping process shows the dissolution of freshly deposited Li in the second stage (from 220 to 275 s) and the old Li that is close to the electrode surface and mixed with the SEI that are oxidized in the second stage (from 275 to 300 s). The removal of the freshly deposited Li is much easier; therefore, the size decrease in the first stage is much faster than the second stage.

### Progressive and instantaneous nature of Li nucleation

Nuclei are aggregates of atoms that become centers for propagation and growth of the new phase in the electrochemically driven phase formation processes. The classical theory of instantaneous nucleation states that the nuclei will generate instantaneously after the nucleation potential η_n_ (*12*, *15*, *52*, *53*). However, in the LMBs, appearance of SEI layer on the electrode surface makes things complicated. The formation and growth of the new Li phase involve a sequence of events, including Li ion complex’s diffusion through the electrolyte, adsorption of solvated Li ion onto the SEI layer and Li ion desolvation, Li ion diffusion and migration through SEI layer, Li ion adsorption on electrode surface, and lastly, the charge transfer and Li ion reduction on the electrode surface to form the Li nucleus. On the basis of theory, the rate of appearance of nuclei on a surface can be expressed as (*23*, *54*).

$$dN/dt=A(N_{0}-N)$$ (2)

where *N* is number density of nuclei on the electrode surface, *N*_0_ is total possible active sites for nucleation, and *A* is the nucleation rate per site. From the Eq. 2, we can calculate the number density of nuclei as function of time can be given by

$$N=N_{0}[1-\exp(-At)]$$ (3)

When the depletion of nucleation sites is very fast (*A* ≫ 1/*t*), the Eq. 3 will be reduced to *N* = *N*_0_. This means that the number of Li nuclei will be a constant throughout the reaction and all the nucleation sites will be occupied with the Li nuclei at the beginning of the reaction instantaneously. Therefore, the constant number of the nuclei on the surface is the indication of instantaneous nucleation formation. On the other hand, if the nucleation rate is relatively slow, then the Eq. 3 will be reduced to *N* = *N*_0_*At*. In this situation, the Li nuclei will grow on the electrode surface progressively or linearly, which is the characteristics for the progressive nucleation (*54*).

The Li nucleation on Cu electrode shows the nature of both progressive and instantaneous nucleation. RIM allows us to image the entire nucleation process; therefore, we know the exact appearing time for each Li nucleus. Figure 3D shows that the nucleation density extracted from the individual image frame changes over time. We can see that, in the early stage of nucleation formation period (from 65 to 80 s, the blue highlighted region), the nuclei density increases linearly with time, which is the characteristic of progressive nucleation formation. We have extracted the nucleation rate *A* from the simple linear fitting (solid blue line is the fitted curve in Fig. 3D) as *A* ≈ 0.594 × 10^4^ mm^–2^. We believe that this is due to the appearance of the SEI layer, which will slow down the Li ion diffusion and make the initial nuclei formation process much longer. After the initial nucleation process (after 80 s), the nucleation density is relatively stable, which shows the nature of instantaneous nucleation. This is because all the possible nucleation sites (*N*_0_) has been occupied. After the initial nucleation, little fresh Li embryos are generated, and the Li ions would deposit on the existing nuclei sites, since they are thermodynamically favored.

### Progressive and instantaneous Li nucleation in different electrolytes

We study the Li nucleation processes in the different electrolytes to further demonstrate the progressive-instantaneous nucleation process of Li. First, the Li nucleation dynamics in the electrolyte of 1 M LiPF_6_/PC with 50 ppm (parts per million) of water as the additive is measured using the RIM. A small amount of water molecules has been used as an additive in the 1 M LiPF_6_/PC electrolyte, which will lead to a high-quality LiF-rich SEI layer and more uniform Li nucleation (*55*). Recently, we used the RIM to study the SEI layers in the electrolyte of 1 M LiPF_6_/PC with 50 ppm of water and found that the LiF layer is much thicker when we have 50 ppm of water as an additive (*43*). Therefore, we expect to see more instantaneous nucleation in this situation. In our experiment, we did observe that the nucleation process in 1 M LiPF_6_/PC with 50 ppm of water is much faster.

Figure S7 shows the nucleation process in 1 M LiPF_6_/PC with 50 ppm of water additive. First of all, the SEI layer formation process is much longer (from 0 to 150 s) when the electrolyte has 50 ppm of water comparing with the electrolyte that does not have 50 ppm of water (Fig. 3A). This indicates a thicker SEI layer on the surface (*43*). After the formation of the SEI, nuclei quickly formed on the electrode surface in ~10 s (blue shaded region in fig. S7), and the progressive process is much faster compared with the 1 M LiPF_6_/PC without 50 ppm of water additive, which is shown in Fig. 3D (the Li nucleation formation process is ~25 s). The much faster progressive process is due to relatively uniform localized electrochemical environments regulated by the improved SEI layer when water additive is added. As a result, the nuclei show smaller heterogeneity and quickly generate on the substrate.

We also studied the Li nucleation process (fig. S8) in the 1 M LiPF_6_/[ethylene carbonate (EC) : dimethyl carbonate (DMC)] electrolyte as a control experiment. We found that after the overpotential reaches the minimum value at around 85 s, the Li nucleation starts to form on the electrode surface. The progressive process lasts around 30 s (the blue highlighted region in fig. S8), which is much longer than that of 1 M LiPF_6_/PC with 50 ppm of water additive. This is because of the low quality of the SEI layer, which leads to a nonuniform electrochemical environment and a wider distribution of the Li nucleation process.

### The relationship between Li nucleation and overpotential

The overpotential is the measure of degree of difficulty to form the nuclei. For galvanostatic electrodeposition, the measured voltage response curve could be referred as three different regions (Fig. 2G) (*15*, *51*, *56*, *57*): (i) An initial voltage drops upon the negative polarization applied and could be attributed to the electrochemical decomposition of the electrolyte to produce the SEI, which will serve as a protective layer during the battery operation; (ii) after the initial voltage drop, a sharp voltage peak below 0 V is observed and defined as the nucleation potential η_n_, suggesting the onset of Li nuclei on the substrate; (iii) and then, the voltage will gradually increase and lastly approach a relatively stable voltage plateau, which can be referred as growth potential η_g_, signifying the continuous growth of Li nuclei. Different from the bulk Li metal evolution process, at a current density *i* < *i*_l_ (*i*_l_, the limiting current density), the initial nucleation process is a reaction-limited process, and hence the applied current density and the resulted overpotential play critical roles in determining the very initial Li nucleation dynamics (*30*, *58*, *59*).

Correspondingly, we did not observe any Li nuclei formed on the electrode surface before the potential reaches the minimum value (the nucleation potential η_n_) around 60 s in Fig. 3D, which supports the conventional theory. The nucleation potential represents the maximum kinetic hindrance effects, including the effects from SEI layer, diffusion resistance, lithiophobic Cu surface, and surface energy. The electrode must overcome this potential to start the Li nucleation process. After reaching this potential, Li nuclei start to form on the electrode surface, which will decrease the lithiophobic effect on Cu surface and reduces the surface energy that are needed for Li nucleation. In the experiment, we do find that the overpotential increases (absolute value decreases) quickly to a relatively stable level (blue highlighted region in Fig. 3A) at 100 s. In this time period (blue highlighted region in Fig. 3A), the electrode surface is partially covered with Li nuclei, and the newly reduced Li will be deposited onto both the Li nuclei that are already formed and the electrode surface to form new Li nuclei at the same time. Therefore, because more and more Li nuclei cover the electrode surface, the kinetic hindrance effect due to the surface energy quickly decreases, and the overpotential also quickly increases (blue region in Fig. 3A). After 100 s, the surface is almost fully covered with Li nuclei (Fig. 2D), and the freshly reduced Li will mostly be deposited onto the Li nuclei instead of the bare electrode surface. The hindrance effect caused by the surface energy will be relatively small and stable (yellow highlighted region in Fig. 3A). The nuclei formation rate is mostly zero and sometimes negative, which is because of the merging of two nearby nuclei.

### Spatially resolved overpotential map

Overpotential is widely used to evaluate the kinetic hindrances in the battery system, including ion diffusion resistance in the electrolyte and the SEI layer, and the surface energy for Li nucleation (*11*, *13*, *52*). However, the traditional electrochemical method only provides an average value from the entire electrode surface. On the other hand, because of the localized electrochemical environments, the overpotentials at different locations could be quite different. Because the differences in the individual particle evolution are closely related to the localized electrochemical conditions, the observation of individual Li particle formation dynamics from our RIM makes it possible to analyze the localized overpotentials on the electrode surface.

Figure 3E shows the early-stage nucleation process of 25 Li nuclei as examples to illustrate that different Li nuclei start the nucleation at different time points. This observation indicates the localized electrochemical environment influences the overpotential and determines individual particles’ nucleation. On the basis of the Barton’s model (*11*), the overpotential represents the total hindrance effects of Li nucleation, including the charge transfer resistance, ion diffusion resistance through the electrolyte and the SEI layer, and the interfacial energy. Therefore, the overpotential can be modeled with the equation

$$\eta =\frac{RT}{\alpha F}\ln\frac{i}{i_{0}}+\frac{iRT}{D_{\infty }C_{\infty }F^{2}}r+\frac{2\gamma V}{F}r^{-1}+\frac{iRT}{D_{S}C_{S}F^{2}}r^{2}$$ (4)

where, η is the total overpotential including all the processes mentioned above; *R*, *T*, α, *F*, and *r* are universal gas constant, temperature, charge transfer coefficient, Faraday constant, and radius of the Li nuclei, respectively; *D*_∞_ is the diffusion coefficient of Li^+^ in bulk electrolyte; *C*_∞_ is the concentration of Li^+^ in solution; γ is the interfacial energy between metal and solution; *V* is molar volume of Li metal; *D*_S_ is the diffusion coefficient of Li^+^ through SEI; and *C*_S_ is the Li^+^ ion concentration in SEI.

**Fig. 4.**
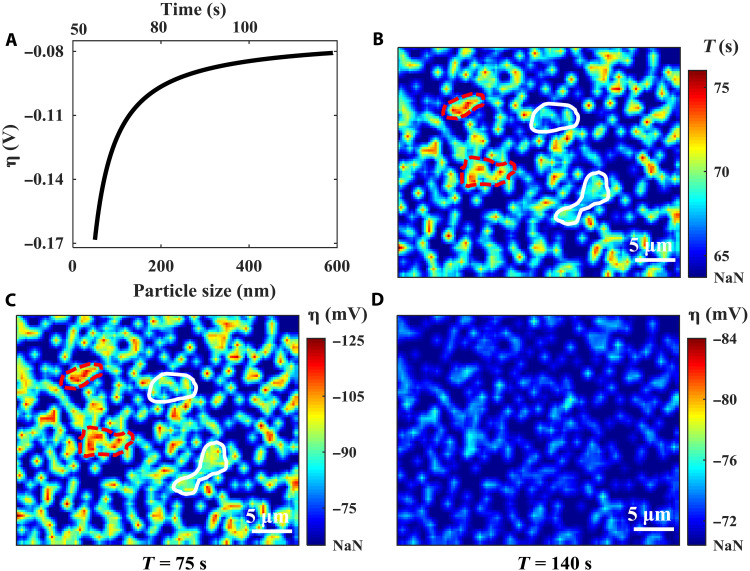
Localized overpotential information. (**A**) Proof-of-concept curve fitting that correlated the total overpotential with the nuclei size. (**B**) The nuclei formation time map. (**C**) The overall overpotential map at the deposition time of 75 s. (**D**) The overall overpotential map at the deposition time of 140 s. “NaN” represents the region without nuclei.

Equation 4 describes the relationship between the nucleus’ size and the overpotential, and the operando RIM provides the individual Li nucleus’ size at different times. Therefore, using Eq. 4 together with the particle size dynamics, we can calculate the overpotential as a function of the particle size [η(*r*)] (see section S9 for details). Figure 4A shows the overpotential calculated from one Li nucleus as an example using Eq. 4. In the experiments, each RIM image is time-stamped, which allows us to correlate the particle size with time. Top *x* axis in Fig. 4A shows the corresponding time. We can see that, starting from 60 s, this Li particle starts to nucleate, and the corresponding overpotential is around −0.17 V. As the Li nucleus grows bigger, the hindrance effects decrease, and the corresponding overpotential obtained from the Eq. 4 also increases (the absolute value decreases; see Fig. 4A). This result has shown that we can get the localized overpotential using the particle size data obtained from RIM.

In addition, we can also identify the position of each Li nucleus in the RIM image. Therefore, the overpotentials as a function of both position and time [η(*x*, *y*, *t*)] can be obtained. We have plotted the overpotential distribution maps at different time points in movie S2, and Fig. 4 (C and D) shows snapshots of the overpotential distribution at two time points. The overpotential distributions are not uniform across the electrode surface. Some regions show more negative overpotentials (red dashed circles in Fig. 4C) compared with other regions (white solid circles in Fig. 4C). This is because the localized electrochemical environment will influence the SEI’s quality and dictate the hindrance effect of the Li nucleation. Bigger hindrance effect will lead to a more negative overpotential and a later nucleation onset. Figure 4B shows the nucleation formation onset map (see section S5 for more discussion). Comparing the overpotential maps (Fig. 4, C and D) and the onset map (Fig. 4B), we can find that, for most of the cases, the larger overpotential delays the nucleation onset (marked by red dash line in Fig. 4, B and C). On the other hand, the particle that appears much earlier is typically associated with a smaller overpotential (marked by white solid line in Fig. 4, B and C). This is expected, since a smaller overpotential indicates a smaller energy barrier for a nucleus to form on the solution/solid interface. The similarity between the overpotential map and the time map demonstrates the feasibility of using the proposed RIM to analyze the initial localized electrochemical information of the substrate, which is critical for controllable electrodeposition that can further promote the performance of electrochemical cells.

## DISCUSSION

We have developed and used an operando RIM to image the entire Li nucleation dynamics with high spatial and temporal resolution. The system enables us to monitor and track the individual Li nuclei’s sizes and locations continuously throughout the deposition and stripping processes. Using the system, we find that both growth and stripping processes of Li nuclei show two distinct stages. For Li deposition, the first stage is correlated to the Li nuclei’s formation and growth until they cover almost the entire electrode surface. The second stage is related to the continuous Li growth. The stripping process shows the dissolution of freshly deposited Li and old Li that is close to the electrode surface and mixed with the SEI. In addition, there are no Li nuclei generated before the overpotential reaches its minimum value and the formations of the initial Li nuclei are not at the same time, which strongly indicates that the localized surface electrochemical environments, including SEI, ion concentration, and surface energy, will determine the Li nucleation and growth. We successfully extracted the localized overpotential map using the particle size dynamics obtained from the RIM and the Barton’s model. The distribution of overpotentials at different locations reflects the heterogeneous surface environments.

## MATERIALS AND METHODS

### Operando RIM

The operando RIM was built on an upright reflection microscopy (Olympus BX50) in an Ar-filled glove box (with H_2_O and O_2_ level less than 0.5 ppm; MBRAUN), and the three-electrode cell was integrated with a reflection optical microscopy. The Li deposition was operated in an organic liquid electrolyte with 1.0 M LiPF_6_ dissolved in PC solvent, which has a relatively high refractive index (around 1.4 in our case) compared with air (~1.0) and water (~1.33). The refractive index mismatch between the electrolyte and air will lead to severe distortion and blur the optical images. To minimize this effect, a special 20× multi-immersion objective (Applied Scientific Instrumentation) was used. The multi-immersion objective can match a wide refractive index range from 1.33 to 1.56, which completely meets the imaging requirements in the electrolytes that are commonly used in Li-based batteries and therefore enables clearly imaging of evolution dynamics of Li nucleation. The numerical aperture of the objective is around 0.68 in the electrolyte that has a refractive index of 1.415. The incident light is mainly distributed around 600 nm (see more discussions in section S10). The spatial resolution of the RIM is defined by the diffraction limit (~300 nm), and the temporal resolution is defined by the camera frame rate. Because the Li nuclei evolution dynamics change a lot at different current densities, the frame rate that we chose would change accordingly to balance the memory volume and critical dynamic information, but it can be ramped up to 1000 frames per second if necessary. A one-sided cell system was designed to enable the light path, with a hollow cylindrical reservoir placed on the substrate and sealed with epoxy glue to hold the electrolyte. The Li nucleation was triggered with an electrochemical workstation (CHI660E, CH Instruments Inc.) and stayed within a kinetically limited regime (*58*, *60*) (see section S12 for details). During the experiments, the detailed Li nuclei evolution process was collected via the reflection microscopy and recorded by a charge-coupled device camera (Pike, F-032C monochrome). The recorded images were then synchronized with the corresponding potential response using a data acquisition card (National Instruments, USB-6009) to realize real-time analysis (see section S3 for details).

### Materials

The Cu film substrate was fabricated via thermal evaporation with a thickness around 30 nm on a 150-μm-thick glass slide (refractive index: 1.5; see section S11 for more discussions). Li metal foil was purchased from Sigma-Aldrich, and the surface was cleaned by doctor blade before use inside the Ar-filled glove box. LiPF_6_ and PC at battery grade were purchased from Gotion Inc. and used without further purification. The electrolyte of 1.0 M LiPF_6_ in PC was prepared inside the Ar-filled glove box where the oxygen and moisture contents were less than 0.5 ppm. The H_2_O content in this electrolyte was about 13 ppm, measured by Karl Fisher titration. The Cu substrate was immersed in the 0.1 M HCl for 1 min to remove the Cu oxide layer, then cleaned with deionized water and acetone sequentially to remove the HCl residue, and lastly dried under vacuum in the antechamber of the glove box.

### Electrochemical tests

A three-electrode system was performed in this study, where the Cu film substrate worked as the WE and Li metal foil worked as the CE and the RE, respectively. A hollow cylindrical reservoir with the diameter of 2 cm and height of around 1.2 cm was placed and sealed on the Cu substrate using epoxy glue. The electrochemical conditions were applied with CHI660E (CH Instruments Inc.), with different current densities and specific amount of deposition capacities. When investigating the influence of deposition capacity on the Li nucleation process, the constant current density with different deposition capacities were applied, and when studying the effect of current density on the nuclei evolution, different current densities with the same deposition capacity were conducted.

## Supplementary Materials

### This PDF file includes:

Supporting Discussions Sections S1 to S12 Figs. S1 to S10 Legends for movies S1 to S3
