# Supplementary material for: Progressive and instantaneous nature of lithium nucleation discovered by dynamic and operando imaging
Source: Sci Adv. 2023 May 24;9(21):eadg6813. doi: 10.1126/sciadv.adg6813 (PMC10208563; doi:10.1126/sciadv.adg6813)
Supplement: Supplementary file 1 — Supporting Discussions Sections S1 to S12 Figs. S1 to S10 Legends for movies S1 to S3 [file sciadv.adg6813_sm.pdf]

Science Advances
AAAS

Supplementary Materials for

Progressive and instantaneous nature of lithium nucleation discovered by dynamic and operando imaging

Guangxia Feng *et al.*

Corresponding author: Wu Xu, wu.xu@pnnl.gov; Xiaonan Shan, xshan@central.uh.edu

Sci. Adv. **9**, eadg6813 (2023)
DOI: 10.1126/sciadv.adg6813

The PDF file includes:

Supporting Discussions
Sections S1 to S12
Figs. S1 to S10
Legends for movies S1 to S3

Other Supplementary Material for this manuscript includes the following:

Movies S1 to S3

Supporting Discussions

1. *Recognizing Li nuclei from background noise*

Due to the scattering effect, when Li nuclei form there will be black spots appearing on the substrate. And we detect each individual particle through optical intensity contrast. However, it will be difficult to recognize very initial nuclei from the background, since the nuclei size is only several tens of nanometer, and the scattering signal is very small. In addition, the electronic pixel noise and drift of the imaging setup further cause trouble for us to detect all the nuclei and monitor their growth from the very beginning. To differentiate signal from Li nuclei and clean substrate, we conducted a control experiment and calculated the optical signal fluctuation from background. A test run before experiments will be performed to make sure the drift of optical system is minimized. Then in the control experiment, no external electrochemical conditions were applied, while the optical images of the observation region were kept recording. Thus, the obtained optical signal variation only responses to the background noise introduced by system instability and pixel noise. Smoothing along time was conducted to further mitigate setup drift and spatial smooth (pixel area: 2 *2) was also performed to reduce the pixel noise fluctuation (**Fig. S1A**). After that, the optical intensity fluctuation distribution was plotted, as shown in **Fig. S1B**. According to statistical analysis, intensity change caused by the background noise is within ±0.05%, indicating that detected optical signals that are larger than 0.05% can be attributed to nuclei formation disturbance.

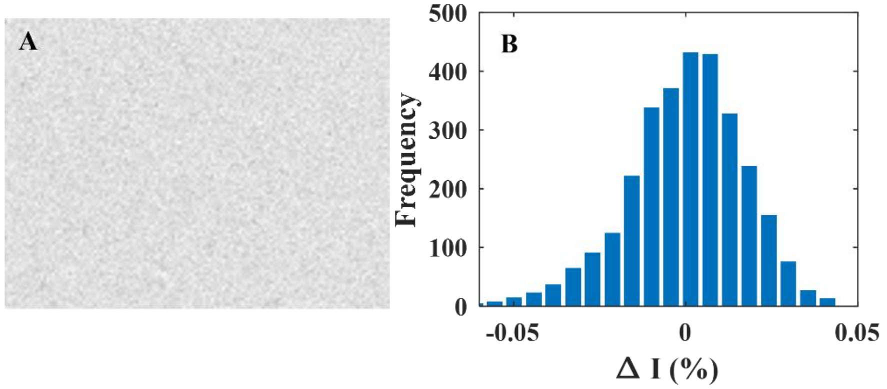

Fig. S1 | Optical signals from background. (**A**) Optical image of electrode surface without nuclei formation. (**B**) Optical signals from the clean electrode surface.

2. *Particle size calibration*

The light scattering is an inexpensive and convenient technique to determine the size and shape of nanosphere and nanorod particles. It has been used to assess haze intensity and particle size for a long history. In our former work, we have adopted this method and experimentally fitting corresponding curve and equation to predict the relationship between the scattering light intensity and Au particle size. To predict the particle size of Li nuclei more precisely, we further extended this method with Li particles that were electrochemically deposited on Cu substrate. To get the relationship curve, we first performed the electrodeposition of Li on the working electrode, which would result in differentiable Li nanospheres on Cu substrate (**Fig. S2A**). For each individual particle, a specific x*x pixel area around the center of the particle location (usually 4×4 in our case) would subtract its blank neighborhood with the same pixel size and then divided by the average intensity of the entire image to obtain the scattering light intensity change causing by particle deposition. For SEM measurement, the substrate was immersed and cleaned with dimethyl carbonate (DMC) for three times and dried in Ar. Then the practical particle size was characterized with SEM. Most of the particles imaged with RIM (**Fig. A2A** and **C**) can be found with the SEM (**Fig. S2B** and **D**), indicating the sensitivity of the RIM to detect early-stage Li nuclei formation. After the information about the practical particle size and corresponding intensity change caused by particle was obtained, the correlation curve and calculation equation could be fitted, as shown in **Fig. S2E**. Thus, once the optical intensity change information was acquired, we could predict the corresponding particle size. One thing we should note is that when the particle is too large, our fitting curve may no longer be used. The analytical data and figures related to the nuclei size information were all based on the fitting curve in **Fig. S2E**. Combining the statistical data from clean substrate (**Fig. S1B**) and the fitting results (**Fig. S2E**), the theoretical particle size resulting from the background noise is calculated to be ~ 30 nm, demonstrating that after the particle growing larger than 30 nm, RIM can differentiate the particles from the background noise and indicating the high sensitivity of our characterization method to detect Li nuclei.

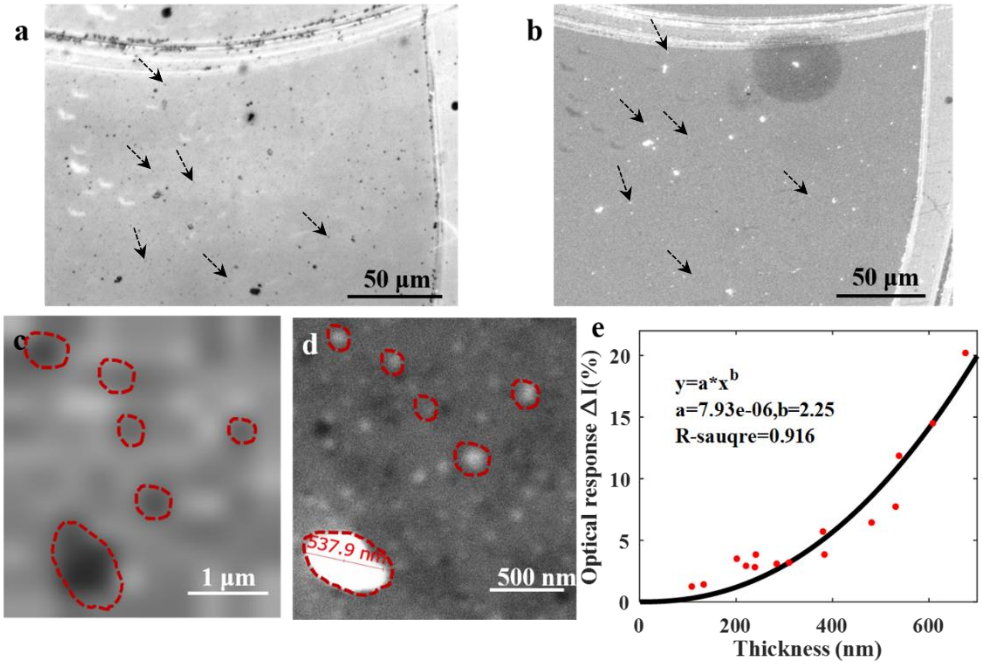

Fig. S2 | Particle size calibration. (**A**) Optical image of deposited Li nuclei, (**B**) SEM image of the same region imaged with RIM. Black arrows mark Li particles that can be easily recognize in both optical image and SEM image. (**C**) Differential image and zoom in region show the individual Li particles. (**D**) Zoom in image obtained with SEM at the same region with (**C**). Red circles mark the deposited Li nuclei on the substrate. (**E**) Conversion curve to translate optical response to particle size.

3. <u>Optical imaging and electrochemical condition synchronization</u>

The Li nucleation process was recorded with the charge-coupled device (CCD). The camera was connected to a computer outside the glovebox with the feedthrough cable, and the camera and the potentiostat were controlled by the same computer. The recorded images were synchronized with the applied electrochemical potentials using a Labview card (National Instruments USB-6009). To ensure that the entire Li nuclei formation process was completely recorded, the optical imaging would record firstly and then applied the electrochemical conditions to trigger the evolution of Li nucleation. By utilizing the Labview card, the optical signals and images from the camera, and the current and potential signals from electrochemical workstation could be accurately synchronized in real-time. Generally, the data acquisition card can collect the electrochemical signal data stream and the CCD imaging pulse signal at the same time. Then with a Matlab code, we can precisely find the specific time when the electrochemical condition is implemented and associate each image frame with its corresponding practical electrochemical information. The synchronization between the optical images and the electrochemical information enables the real time analysis of the Li nucleation dynamics.

4. Nucleation at the very early stage

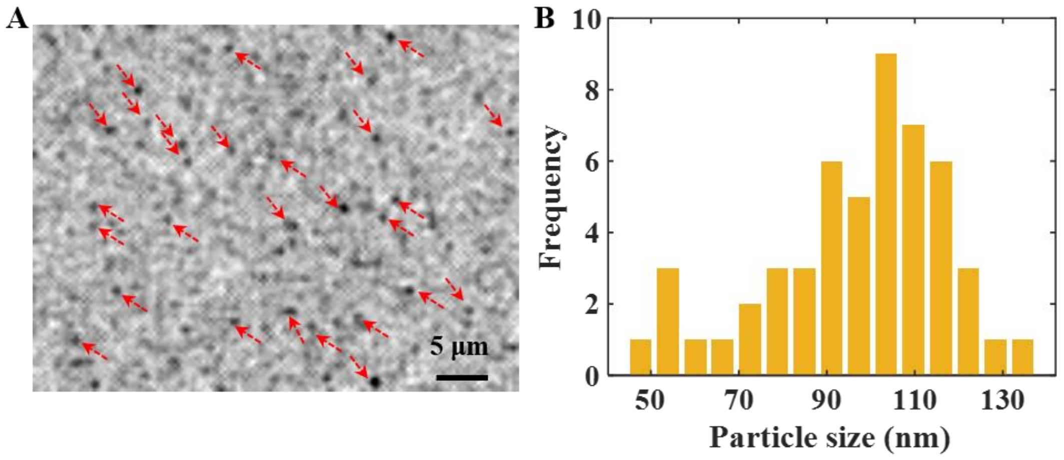

Fig. S3 | Very initial nuclei that formed on the substrate. A, Optical image of nuclei at ~ 60s**. B,** Nuclei size distribution at time ~ 60s.

5. Nuclei density and formation time calculation

The generated Li^0 nuclei will scatter more optical light, so they appear as black spots on the substrate and lead to the decrease of the overall reflected optical signal. Thus, for a given nucleation optical image, the traditional way to detect the formed nuclei on the substrate is setting a threshold, wherever the intensity value smaller than the threshold should be regarded as the nuclei formation spots, otherwise should be recognized as free substrate background. However, before the nuclei formation, the solid electrolyte interphase (SEI) would accumulate firstly, which would cause the optical response to increase or decrease, influencing the baseline of the overall optical response, making it difficult to set a constant threshold to detect the formed nuclei. On the other hand, as the deposition capacity gradually increased, some of the nuclei would merge with each other, leading to the decrease of the complete nuclei amount. To solve these problems, we finally chose the image frame that before nuclei merger as the overall nuclei map and set a threshold to detect all the nuclei spots (as shown in **Fig. S4**), these detected spots were then referenced as the addressing map to determine the earliest time they formed on the substrate. For each nucleus, the first image frame where the optical intensity decreased by more than 2% compared to the initial substrate would be identified as the time point that the nucleus appeared. Therefore, by calculating the nuclei generation dynamics along with time and divided by the entire substrate area, the nuclei density information could be obtained for further analysis.

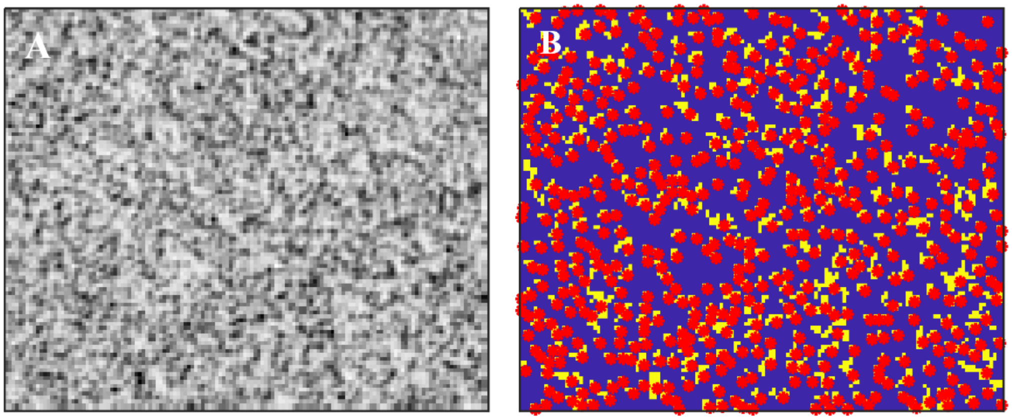

Fig. S4 | Nuclei detection. (**A**), Optical image of Li^0 nuclei formed on the Cu substrate. (**B**), Detect the formed nuclei by setting a threshold. The yellow regions signify the regions that have intensity value smaller than the threshold and the red makers label the individual nuclei.

6. Li nuclei growth and size conversion

As discussed in section 4, for each Li nucleus, the first image frame where the optical intensity decreased by more than 2% compared to the initial substrate would be identified as the time point when the nucleus first appears. Before that, optical signal variations were introduced by SEI formation and the entire nuclei growth and dissolution are regarded to be included in the remaining recording optical images. The optical intensity of a randomly chosen Li nucleus was plotted along time (**Fig. S5A**). The result showed as electrodeposition proceeded, more optical signal would be scattered by the Li nucleus and the detected intensity kept decreasing. After reversing external current density, the detected optical signal gradually increased but did not exactly go back to its original value. Utilizing the conversion curve in **Fig. S2E**, the size of the Li nucleus was calculated and shown in **Fig. S5B**. With continuous applied deposition current, Li nucleus was observed to gradually grow into large size and scattered more incident light. After the reversal of current, the nuclei would shrink which was accompanied by increasing optical intensity. The nucleus still existed at the end of dissolution, as evidenced by the optical signal that did not return to its initial value (**Fig. S5A**).

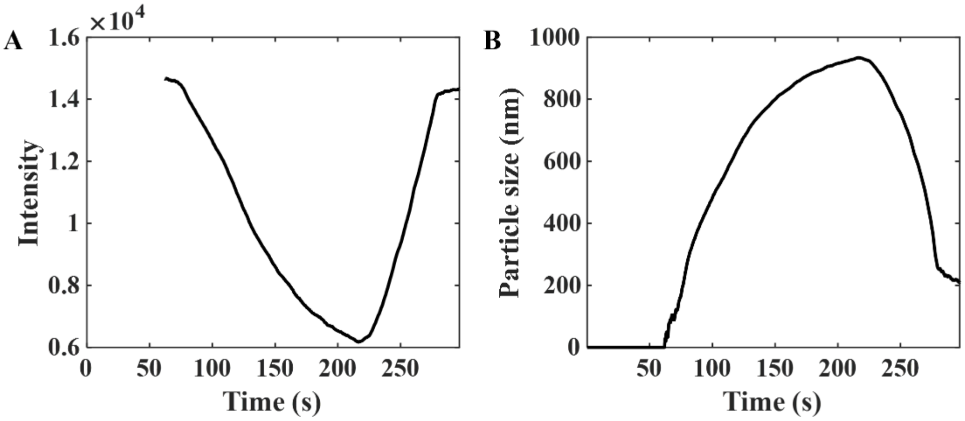

Fig. S5 | Individual particle growth process. (**A**). Optical signal variation during the growth process of Li nuclei, (**B**) Particle size evolution process which is calculated from optical signal.

7. Li nucleation at different current density

Keeping the deposition capacity constant, the Li nucleation behavior was investigated at various current densities. At a relatively small current density of 0.02 mA cm^{-2}, the Li nuclei were non-uniformly formed and sparsely distributed on the electrode surface (**Fig. S6A**). As a comparison, when the deposition current density increased to 0.1 mA cm^{-2} (**Fig. S6B**), a significant increase in particle count could be observed. Further increasing the deposition current density to 1 mA cm^{-2}, a considerable amount of Li nuclei formed and spread uniformly on the electrode surface (**Fig. S6C**), indicating that the generated nuclei is closely related to the applied electrochemical condition and the initial nucleation is a reaction-limited process. The calculated nuclei area density shown in **Fig. S6D** displays that the nuclei areal density is around 0.02 counts µm^{-2} at the current density of 0.02 mA cm^{-2}, while at 1 mA cm^{-2} the areal density reaches around 0.3 counts µm^{-2}, demonstrating an obvious positive correlation between the current density and the nuclei density. Such phenomenon has been previously observed by other researchers and they claimed that the nuclei density was positively related to the overpotential. To correlate the current density with overpotential, the voltage profiles of the corresponding galvanostatic deposition were plotted. From **Fig. S6E** we can find that both the nucleation overpotential (η_n) and the nuclei growth overpotential (η_g) increase as the increment of the applied current density. The nucleation overpotential (η_n) increased from around -100 mV at 0.02 mA cm^{-2} to around -450 mV at 1 mA cm^{-2}, and the growth overpotential (η_g) also increased from around -50 mV at 0.02 mA cm^{-2} to around -300 mV at 1 mA cm^{-2}, which are rational according to the Butler-Volmer electrode kinetics equations. Another thing we should notice is that the nuclei growth

potential is always smaller than the nucleation potential, proving that it is more favorable for Li to deposit on the already existed nuclei rather than to form new nuclei sites due to the higher energy barrier for the Li metal phase to form on Cu substrate.

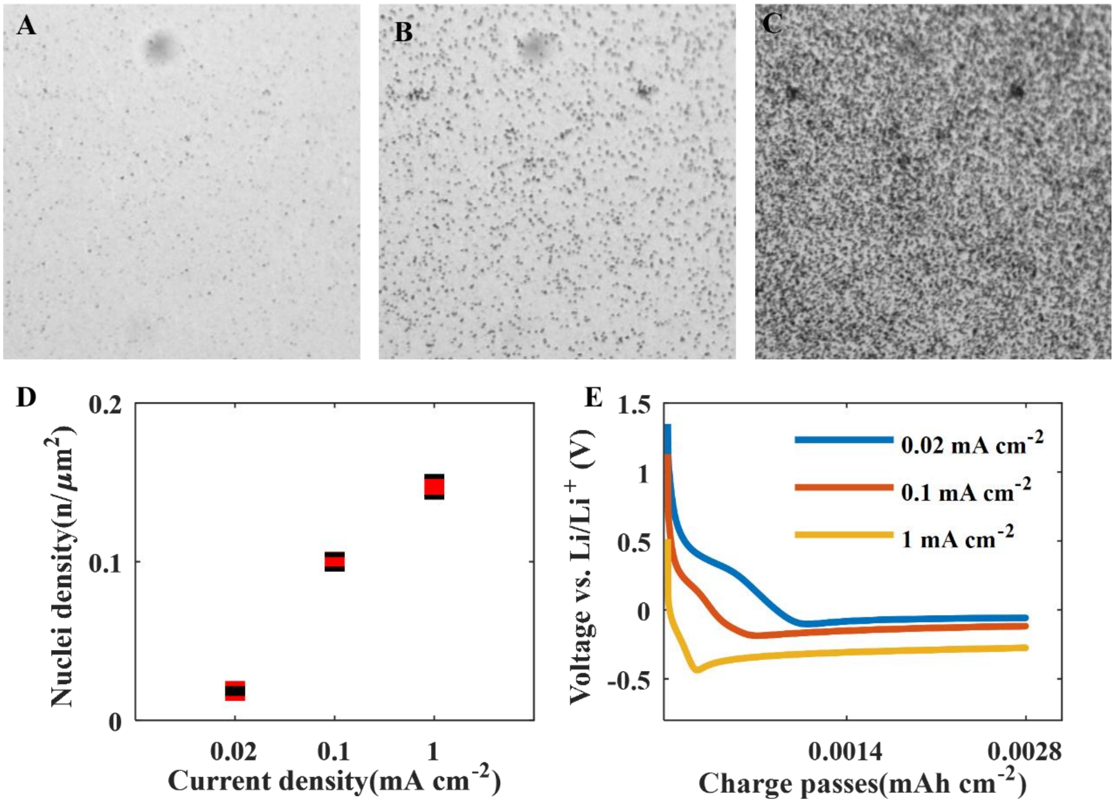

Fig. S6 | Li nucleation at different current density. (**A-C**). In-situ optical images of Li deposited on the working electrode at current density of (**A**) 0.02 mA cm^{-2}, (**B**) 0.1 mA cm^{-2} and (**C**) 1 mA cm^{-2}, for a total deposition capacity of 0.0028 mAh cm^{-2}. (**D**). The nuclei areal density at different current densities. (**E**). Potential profiles of galvanostatic Li deposition under different current densities for a total areal capacity of 0.0028 mAh cm^{-2}.

8. *Progressive-instantaneous characteristic of nucleation process*

To confirm the progressive-instantaneous characteristic of Li nucleation process, Li deposition under current density of 0.1 mA cm^{-2} in the electrolytes of 1 M $LiPF_6$ in PC with 50 ppm water additive and 1 M $LiPF_6$ in EC/DMC were performed.

Li deposition in electrolyte 1 M $LiPF_6$/PC with trace amount of water as additive was performed because water will lead to a high quality LiF SEI layer and more uniform Li nucleation. Recently, we used the RIM to study the SEI layers in the 1 M $LiPF_6$/PC with 50 ppm of water, and found that the LiF layer is

much thicker when we have 50 ppm of water as additive. Therefore, we expect to see more instantaneous nucleation in this situation. In the experiment, we did observe the nucleation process in 1 M $LiPF_6$/PC with 50 ppm water is much faster. **Fig. S7A** shows the potential response curve and the corresponding optical signal change as a function of time. Solid-electrolyte interphase (SEI) will form before Li atoms aggregate and generate nucleus. Therefore, we observed two potential steps at ~1.8 V and ~0.3 V from the potential response curve (red curve in **Fig. S7A**) that are related inorganic-enriched SEI generation and organic-enriched SEI formation, correspondingly. After SEI formation and reaching the nucleation potential, Li nuclei began to generate on the substrate. Obviously, the nuclei progressively formed as deposition capacity increased, indicating the very initial progressive feature of nucleation which could be caused by substrate defects and the heterogeneity of SEI on the electrode surface. However, the generated nucleation process in 1 M $LiPF_6$/PC with 50 ppm water additive demonstrates a much faster progressive growth stage (~ 10 s, marked with blue background in **Fig. S7**) compared with the 1 M $LiPF_6$/PC without water additive, which is shown in Fig. 3D (The Li nucleation formation process is ~25 s), indicating the localized overpotential is relatively uniform when there is water additive and therefore the nuclei show smaller heterogeneity and the nucleation sites will be quickly stabilized. After deposition time of ~ 160 s, although deposition capacity continuously increased, the nuclei densities were relatively stable, signifying very little active sites for newly formed Li nuclei and the total number of nuclei on the electrode was almost constant after the early-stage formation which could be regarded as features of instantaneous nucleation mode.

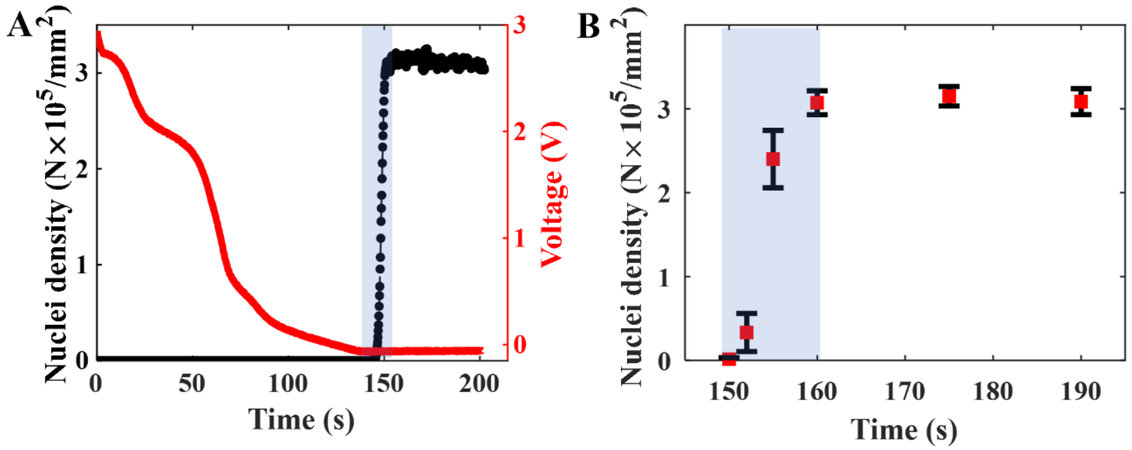

Fig. S7 | Nucleation process in electrolyte 1M $LiPF_6$/PC with 50 ppm water additive. A, Li nuclei density evolution (black dots) along with deposition time and potential (red curve). Blue background marks the early-stage progressive nucleation formation. **B**, The areal density of nucleated particles at different time points.

We also studied the Li nucleation process in the 1 M $LiPF_6$/(EC:DMC) electrolyte as the control experiment (**Fig. S8**). It was found that after the overpotential reaching the minimum value at around 85 s, the Li nucleation starts to form on the electrode surface and the progressive process lasts around 30 s (blue highlighted region in **Fig. S8**). This is because heterogeneous electrochemical environments that are introduced by either poor quality SEI or substrate defects.

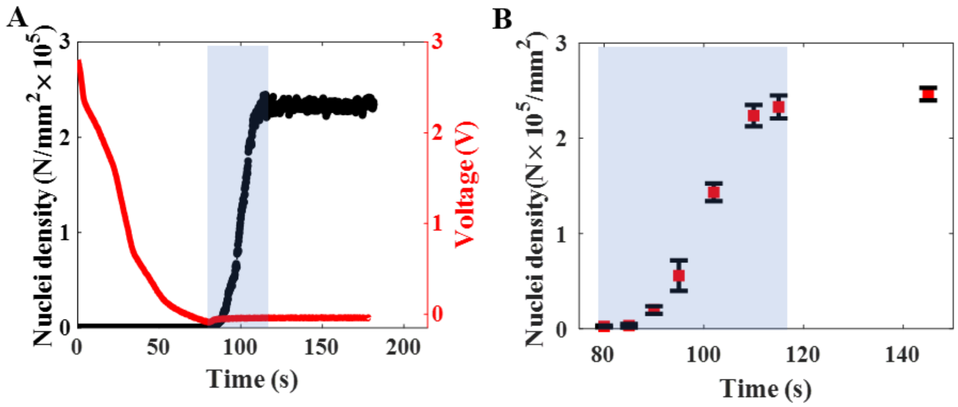

Fig. S8 | Nucleation process in electrolyte 1M $LiPF_6$/(EC:DMC). A, Li nuclei density evolution (black dots) along with deposition time and potential (red curve). Blue background marks the early-stage progressive nucleation formation. **B**, The areal density of nucleated particles at different time points.

Li nucleation on three different electrolytes supports our conclusion that Li nucleation possesses progressive-instantaneous nucleation characteristics and demonstrates the influence of electrochemical environment on the nucleation process.

9. *Localized overpotential and charge transfer overpotential*

As we have mentioned, traditional techniques usually measure the averaged overpotential value from the entire electrode, but the localized information is critical since it reveals the heterogeneity of the localized environment that will play an important role in determining the growth morphology and structure of Li. Due to the powerful in-situ and wide field of imaging characteristics of the proposed RIM, it becomes possible to probe localized overpotential by analyzing the individual particle growth dynamics. Barton's model describes the correlation between nuclei particle size and overpotential, which represents the total hindrance effects of Li nucleation, including the charge transfer resistance, ion diffusion resistance through the electrolyte and the SEI layer, and the interfacial energy. We use the measured overpotential and the averaged Li nuclei growth dynamics to fit and obtain the four constants in equation (4), including $\frac{RT}{\alpha F}$,

$\frac{RT}{D_\infty C_\infty F^2}$, $\frac{2\gamma V}{F}$, and $\frac{RT}{D_S C_S F^2}$. At the very initial stage of the reaction, the electrochemical environments (before SEI formation and nucleation) are uniform across the electrode surface. After applying the external potentials, the electrochemical environments evolve locally and the growth dynamics is different for each individual Li nucleus. The variations of localized electrochemical environments will converge into the localized overpotential variations and influence the Li nucleus growth rate. RIM provides us with the Li nuclei size information over time, which allows us to extract the localized overpotential using the Barton's model with the fitted constants from equation (4).

Combining growth dynamics of individual Li particle obtained from RIM and the potential curve during galvanostatic deposition, each individual Li particle will produce unique charge transfer overpotential, nucleation surface energy, and ion diffusion overpotential in electrolyte and SEI. Charge transfer overpotential, also referred as activation overpotential, is crucial for evaluating the initial localized electrochemical information of the working electrode. By extracting the first term of the Barton model equation, a charge transfer overpotential map can be achieved. As shown in **Fig. S9**, the charge transfer overpotential map reveals obvious difference across the electrode surface, with some regions have a relatively smaller charge transfer overpotential while other regions own a larger overpotential value, and the maximum difference is around 40 mV. The difference shown in charge transfer overpotential map also supported the very initial appearance time difference of the Li particle.

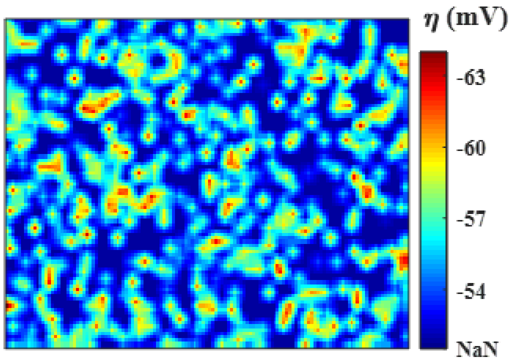

Fig. S9 | Charge transfer overpotential map. It is obtained by extracting the first term of the Barton model equation.

10. <u>Incident optical source and potential influence on nucleation behavior</u>

In this study, a mercury lamp light source was used with a bandpass filter (~10 nm band width at around 600 nm) in front. This will provide us with a relatively narrow band incoherent light source for interference imaging. The incoherent light source will minimize the interference fringes that will significantly affect the

imaging quality. The spectrum after passing through the filter has been shown in the (1) (Supplementary Fig. 5). To understand the effect of light source to the reaction, we measured the power of the light that reach the electrode surface, which is ranging from 0.3 mW ~ 0.78 mW. And the photon density at the field of view is calculated to be 3×10^9 ~ 1.15×10^9 photons/μm^2. The reflectance of optical intensity will be ~ 70%, neglecting the transmittance for Cu substrate, the absorbed optical intensity will be ~ 30%. Assuming all the absorbed optical light will be converted into heat in the imaging system, it will only cause temperature increase at around 0.0027 ~ 0.007 °C. Therefore, the intensity of the light will not influence the nucleation process.

11. Surface feature of working electrode surface

After cleaning, the surface roughness of the Cu substrates was mapped with AFM. **Fig. S10A** shows the morphology of a cleaned Cu substrate, and the surface roughness is calculated to be ~ 2 nm. In addition, we randomly selected a line across the imaging region and plot the corresponding profile in **Fig. S10B**, the height variation is close to ~ 1.5 nm.

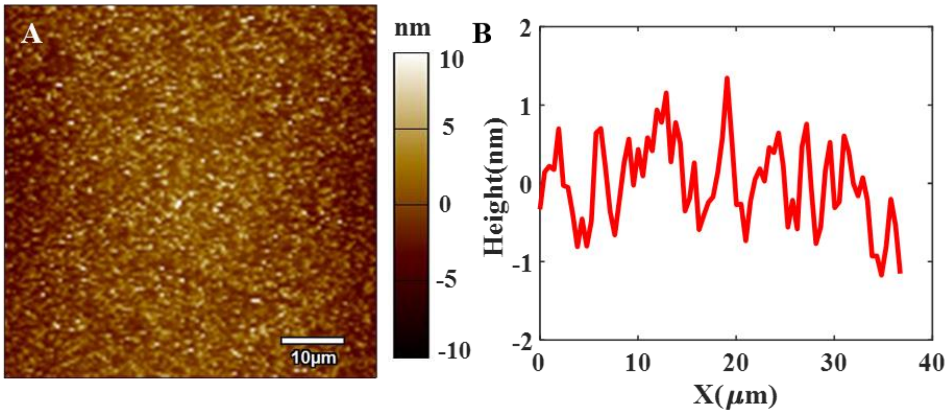

Fig. S10 | Surface roughness of Cu working electrode. A, AFM imaging of the clean Cu substrate. **B**, Random cross section profile of the Cu surface.

We believe the surface conditions, including roughness, the SEI accumulation, and the electrolyte compositions (we called it localized electrochemical environments) together will influence the following Li nucleation process. Therefore, we calculated the localized overpotential map in Fig. 5 in the main manuscript, which reflects the total effects from the localized electrochemical environments including both the SEI and initial surface defects and reveal the correlation between the nucleation behavior and electrochemical environments difference.

12. *Influence of cell geometry on the inhomogeneity of electrochemical environments*

The size of working electrode is 4 mm × 10 mm, and the counter electrode is lithium foil with the size of 4 mm × 6 mm, and the distance between them is around 5 mm. The limiting current density is calculated by $J_{lim} = 2z_c c_0 FD(t_a L)^{-1} \approx 1\ mAcm^{-2}$, where z_c is the charge number of Li^+, c_0 is the concentration of the electrolyte, F is the Faraday's constant, D is diffusion coefficient, t_a is the charge transfer number and L is the distance between electrodes. When the current density $J > J_{lim}$, the local ion depletion and hydrodynamic instability (known as electroconvecton) happen, which leads to the morphological instability and the dendrite growth. However, the current density we applied (0.1 mA cm^{-2}) is smaller than the limiting current density (1 mA cm^{-2}), therefore during the deposition time the ion depletion may not happen, and the experiments stay within a kinetically limited regime.

Supplementary videos:

Supplementary Video S1: Li deposition and stripping processes under galvanostatic deposition of 0.1 mA/cm^{-2}.

Supplementary Video S2: The evolution of overpotential distribution during the continuous Li deposition process.

Supplementary Video S3: The detailed stripping process in Supplementary Video S1. The stripping was happened under stripping current density of 0.1 mA/cm^{-2}.
